# Hospital Reports—University of Pennsylvania, September 12, 1870

**Published:** 1870-11

**Authors:** J. E. Garretson

**Affiliations:** Lecturer on Surgical Diseases of the Mouth, Etc.


### HOSPITAL REPORTS—UNIVERSITY OF PENNSYLVANIA, SEPTEMBER 12, 1870.

Clinic of J. E. GARRETSON, M.D., Lecturer on Surgical Diseases of the Mouth, Etc.

(REPORTED BY DE FOREST WILLARD, M.D.)

### ANGIONOMA.

GENTLEMEN: I have to present to you this morning, two patients, both infants, who will well illustrate almost the two extremes of a class of tumors known as vascular or erectile growths, or, as often called, *nœvi.*

Upon the cheek of the first you will notice a small, dark spot, scarcely distinguishable, yet sufficient to attract the eye of the anxious patient. It is a "mother's mark," and is an insignificant, harmless, little tumor, requiring but a simple operation. The other, a colored babe, æt. 6 months, has, as you perceive, a tumor upon the cheek, in the neighborhood of the angle of the jaw, and extending into the parotid region, of the size of a small orange, which, as you will soon discover by the history, is a grave and serious lesion, requiring an operation of such magnitude as to place the life of this child in jeopardy.

The mother informs me that this was discovered soon after birth, but was then of small size; that it has been steadily growing, and that for the last few weeks it has increased with such alarming rapidity that she has been sent to us from the country, to ascertain whether there be any hope of operative relief, for she fully understands that the child must die, either from rupture or ulceration of this mass, with its consequent hemorrhage, unless it can be removed.

You will see that this tumor is soft and elastic; that it pulsates strongly, synchronously with ventricular sistole; that its size can be diminished by steady pressure, and that it enlarges whenever the child makes any violent exertion, as in crying, coughing, etc. It is, therefore, an erectile growth—a congeries of dilated bloodvessels; tortuous arterioles and capillaries, bound together by subcutaneous connective tissue; it is a vascular tumor, and belongs to the same class as the nævi.

The color of these vascular growths depends upon the excess in their composition of either the arterial, venus, or capillary element, and this color does not gradually fade out into the surrounding tissues, but is abrupt in its definition, owing to the fact that the tumors are usually supplied by one or two large bloodvessels, while their vascular connection with adjacent parts is but moderate, and the smaller nævi are evidently but a hypertrophy of the radicles of these principal vessels.

In the tumor before us we have one which is largely arterial. It is what is often called an "aneurism by anastomosis," but it is not, properly speaking, an aneurism, but is an enlargement of the terminal branches of these vessels of supply, and is, in fact, an exaggerated nævus, its variation being in degree, not in kind. The same remark will hold good in regard to the venous tumors so often found, which differ only in having an excess of venous dilitation. They may differ widely in degree, but they all belong to one genus,—they are vascular, "bloody" tumors.

For the relief of such a lesion as this, a cutting off of the source of supply would at once suggest itself; but should we cut down and tie the external carotid, we know that the anastomoses of the arteries of the face are so intimate with those of the opposite side, as also with the internal carotid by means of the nasal and other branches of the ophthalmic, that we should not thus be able to check the current; nay, further, should we even ligate the common carotid, we might still have the supply kept up by the circuitous but constant route of the profunda cervicis from the subclavian with the princeps cervicis from the occipital, a branch of the external carotid, a circulation being thus quickly established, and our object defeated, after all the dangers of such an operation. This method, therefore, will not be adopted; but we will take the surer plan: that of excision. This, truly, will be a formidable operation, when we consider how vascular are these parts, even in the normal condition, and the close proximity of such large arterial trunks as you will see by this dried preparation; and I doubt not, ere the excision be completed, we shall be down upon the carotids themselves; yet, with proper care, and due attention to the prompt ligation of all severed vessels, I think we shall be able to remove it without sufficient loss of blood to endanger the child's life.

We shall not, however, endeavor to remove this mass entirely by the knife, but shall first cut away all the surrounding structures, and then throw a strong ligature around its base, thus strangulating the remaining portion, and ridding ourselves of the greatest source of alarming hemorrhage, *i. e.*, the last few incisions which would cut the great vessels supplying it.

When the tumor is small, as in the first case before us, *strang-*

*ulation* is the preferable method, either by encircling the mass as a whole, or, as the skin is somewhat liable to take an erysipelatous inflammation, by first circumscribing it with an incision of the skin, and then directing the ligature into this cut surface. Another method is to pass a double ligature through its base, and tie it in two portions. The second method is best, in my estimation.

Another mode of cure in these small growths is by the action of pressure, either by means of a compress, or by the use of collodion; but this procedure is only applicable to certain situations. Needles, heated to whiteness, have also been passed through the base of these tumors, and have sometimes been followed by cure.

Electrolysis has also been of service in some cases of nævus, but it is limited in its application, and is so slow in its action that patients usually prefer the more rapid method of excision or ligature. The great difficulty to be encountered in the cure arises from the fact that occlusion of a veinule or other radicle seems to have but little influence upon its neighbors, thus necessitating repeated operations until the electrolytic action has directly influenced almost each individual vessel. Moreover, since electrolysis cauterizes the tissues, as well as coagulates the blood, it is evident that a slough must ensue, provided the superficial portion of the skin be much affected, and, if such an occurrence must take place, with its consequent cicatrix, it is preferable to have it occasioned by the more speedy action of a ligature. The slough of galvano-puncture is, however, perfectly devoid of hemorrhage, since it is tardy in its separation, and is remarkable for its extreme dryness.

These objections to its use, however, only apply to nævi which are superficial, or when the skin is implicated. In subcutaneous nævi, the operation possesses the advantages of being more safe and certain than injection, and in cases where no slough is necessitated, we dispense with the scar of an excision or ligitation, that is, provided insulated needles are employed.

A Bunsen or other battery may be used, the number of needles varying with the size of the tumor; but, in all cases, care should be taken not to carry the action beyond the whitish hue, indicative of cauterization.

In regard to the introduction of gas into the circulation by this method, I do not think there is the slightest danger in cases of nævus, though spoken of by Rutherford and other able writers on electro-therapeutics.

In cases of huge nævi, or vascular tumors, like the one before us, this course might be pursued with advantage, especially when excision is hazardous, but I think we shall be able to accomplish our object in perfect safety with the knife and ligature, saving much time and trouble by such a course.

Another mode of removal of nævi is by the use of caustics, such as chlor. zinc, corrosive sublimate, Vienna paste, or the strong acids, and a cure is usually affected by a separation of a slough and healing of the ulcer.

The seton is sometimes used, and its presence occasionally causes sufficient inflammation to obliterate the vessels and leave behind no perceptible scar.

Hypodermic injections were at one time quite in vogue, but owing to several deaths which have occurred from the carrying of clots into the circulation, have fallen into disuse. The substances used were iron, nitric acid, iodine or creasote.

A method practiced by some English surgeons is that of "piece-meal" removal, *i. e.*, picking out the mass, or tearing it away, fragment by fragment, the object being to prevent hemorrhage, upon the same principle as tortion of arteries.

From all these operations, then, we must select the one most suitable to the case upon which we are to perform it, and each will be found applicable to certain localities or conditions; as a rule, however, I prefer the use of the knife to circumscribe the tumor, then to be followed by the ligature.

In regard to the time of operation upon an infant, I will only say that we should always remember that a nævus may spontaneously disappear, or atrophy a few weeks after birth, or it may ulcerate and thus destroy all the unhealthy tissue; still this event is so rare that we need not wait long for its occurrence. The age of the child will not make any great difference. Its condition will, however, vastly influence the result, *i. e.*, a child, who is teething, or has just recovered from measles, cholera-infantum, etc., will be much less likely to bear an operation well, than one who had fully recovered from such a difficulty. Put your patient in a good, sound health, and then operate, no matter what may be its age.

[Two incisions, each two inches in length, and crossing at right angles, were then made through the integument, and the four flaps dissected off the tumor. The knife was then carried through healthy tissue, completely about the mass, each artery being compressed or picked up, as it was divided, so that comparatively little blood was lost. As the deep portions were reached, it became necessary to cease the incisions on account of the size of the arteries, and a strong ligature was then thrown around the base, tightly strangulating the remainder. The wound was dressed with carbolic acid oil; stimulants and anodyne were freely given, and the infant soon rallied. On the fifth day the slough separated, and the flaps, which had before been left loose, were laid in place, and secured by hare-lip pins and the interrupted suture. The cure has been most satisfactory. DE F. W.]

---

DEFORMITY OF THE MOUTH—DIEFFENBACH'S OPERATION.

The next case which I have to show you is Robert C., æt. 11 years; suffering from a deformity of the mouth, resulting from the contraction of a cicatrix of a slough, which slough was one of the many concomitants of scarlatina, a disease which, as you know, may be followed by lesion of almost any structure or portion of the body.

You will perceive, the left angle of the mouth is drawn downward and pulled in, or puckered in such a manner as to give an unpleasant appearance to his face, and he desires us to rectify this condition. This is not a common condition, yet it is sometimes met with from wounds, bruises, etc., and we must study the complications which exist in each case, governing our operation by the extent and character of the contraction.

In looking at a case of this kind, you must bring to bear upon it all your artistic, as well as surgical, skill; you must look at and view it in different positions, until you have decided what alterations are necessary to bring this deformed mouth into a normal condition, with the labial curves and commissures in their usual beautiful symmetry; and, in order to do this, map out in your mind, or, still better, in ink upon the face, the proper outlines of a perfect mouth; then you can arrange your incisions to suit the particular deformity.

In the present instance, the contraction is not so great as is often seen, where the orifice is sometimes so small as scarcely to admit of the passage of the little finger, and is, therefore, comparatively an easy case.

For the relief of this difficulty, our first thought would be to merely incise the commissures to the required extent; but, unfortunately, such a procedure would only be followed by speedy union and greater consequent contraction than before, since it is impossible to prevent union of the fresh surfaces, even though tents, lead, etc., be placed between the edges.

Mechanical dilitation gives severe pain, and does not produce any permanent good.

The only method which has proved of service has been that of Dieffenbach, of Berlin, which is performed by the surgeon first introducing the fingers of one hand into the mouth of the patient, in order to guide and direct the incisions and make the cheek tense, during which time he should stand either behind or in front of his patient, according to the side of the mouth upon which he is to operate, and then inserting the blade of a pair of scissors into the cheek about two lines outside the point which he intends shall be the labial commissure, he cuts inward and upward, dividing all the tissues save the mucous membrane, until he has reached a point in the upper lip, which will, when brought down, fulfill the required conditions. Again, a similar incision is made from the same starting point, but this time toward the lower lip, which, in turn, is divided nearly to the same extent as above; the cuts in each instance being varied to suit the irregularities in the position of the contracted orifice. Next, dissect away the flesh down to the submucous coat, thus leaving but the mucous membrane, which must in its turn be divided by a straight line running out its centre, to a point two lines inside the angle of external incision.

The mucous membrane is now turned over to imitate the normal lip, above and below, on each side, and its edges sutured to those of the skin, thus forming the vermilion border of a new lip.

This operation usually answers admirably, but sometimes, unfortunately, the mucous membrane also participates in the lesion, and the operation cannot be successfully performed; or, again, certain accidents may come between the surgeon and success, and cases will be found where this operation will not cure.

In such cases I am in the habit of modifying, or rather of adding to, or associating a mechanical appliance with these surgical means.

This consists of what might be called a "mouth-stretcher," an instrument made of wood or rubber, with a deep grove around its border, which is slipped between the lips after time has been given for union of the reflected mucous membrane. The lips will be caught and held by the gutter of the apparatus, in the whole of their circumferences, and thus not only is the healing influenced to a desired shape, but undue citratrical contraction is also prevented. This instrument should be worn constantly for a week or two, after which time its use should be continued at night until all contraction has ceased. This apparatus also does more than merely prevent contraction; it may even remedy defects which are the fault of the operator, by compelling the regular healing of the wound, and such mistakes and blunders you will make, unless you carefully weigh each cut, and look at it in various aspects—you must bring all your mechanico-surgico skill into use.

[The incisions were then made as described, the tissues being removed down to the mucous membrane, which was brought over and firmly stitched to the integument. At each step of the operation, the case was viewed, both from a near and distant standpoint, thus rendering the final adjustment a more perfect success. The hemorrhage was but slight, and soon ceased. Cold water dressings were applied, and the boy directed not to exert any strain upon his mouth until union should be well accomplished.—De F. W.]—*Medical and Surgical Reporter.*

